# Moyamoya Disease with Coexistent Hypertriglyceridemia in Pediatric Patient

**DOI:** 10.1155/2016/7974182

**Published:** 2016-10-24

**Authors:** Jacqueline Chan, Fabiola D'Ambrosio Rodriguez, Deepank Sahni, Claudia Boucher-Berry

**Affiliations:** Department of Pediatric, Children's Hospital of the University of Illinois, Chicago, IL, USA

## Abstract

Moyamoya disease is a rare chronic and progressive cerebrovascular disease of the arteries of the circle of Willis that can affect children and adults. It has been associated with multiple diseases, including immunologic, like Graves' disease, diabetes mellitus, and SLE. Hyperlipidemia has been recognized in patients with Moyamoya disease with an incidence of 27–37%. However, no case in pediatric patients has been reported of the coexistence of Moyamoya disease and hyperlipidemia. Here we present a case of a 9-year-old female diagnosed with Moyamoya disease after a stroke with incidental finding of familial hypercholesterolemia. This finding will make our patient a very unique case, since there has not been any reporting of Moyamoya disease and hypercholesterolemia association.

## 1. Introduction

Moyamoya disease (MMD) is a rare idiopathic chronic and progressive steno-occlusion of bilateral intracranial arteries of the circle of Willis that develop in children and adults. It is characterized by collateral vessels seen on cerebral angiography. An immunologic basis has been suggested for this disease and recent reports have noted an association between Moyamoya and autoimmune diseases including Graves' disease, diabetes mellitus, and Systemic Lupus Erythematosus. Analysis of the comorbidities may be helpful in determining the pathogenesis of Moyamoya [1]. Usual age of onset of MMD in pediatric is after 5 years and progresses more rapidly than in adults [2].

MMD is mainly found in East Asia, especially in Japan and South Korea [3–6]. Because MMD is extremely uncommon in the western countries, no systemic surveys have been conducted in either Europe or North America although there have been some sporadic reports [7, 8].

Familial combined hyperlipidemia (FCHL) is a dominantly inherited hyperlipidemia that occurs in at least 1% of the adult population and is responsible for 10% of premature coronary artery disease. Several metabolic defects apparently are associated with the FCHL phenotype. Most commonly, excess production of very low density lipoprotein apolipoprotein B can be demonstrated [1, 9].

Hyperlipidemia has been recognized in patients with Moyamoya disease with an incidence of 27.7% in a study done in Mayo Clinic Minnesota 1979–2011 and 37.3% in a study done in Japan 2001–2011 [1, 10]. However, no study has been documented in a pediatric patient with abnormal lipid profile with concurrent Moyamoya disease. We report a case of a 9-year-old female with Moyamoya disease and coexistent hyperlipidemia.

## 2. Case Presentation

**Figure 1:**
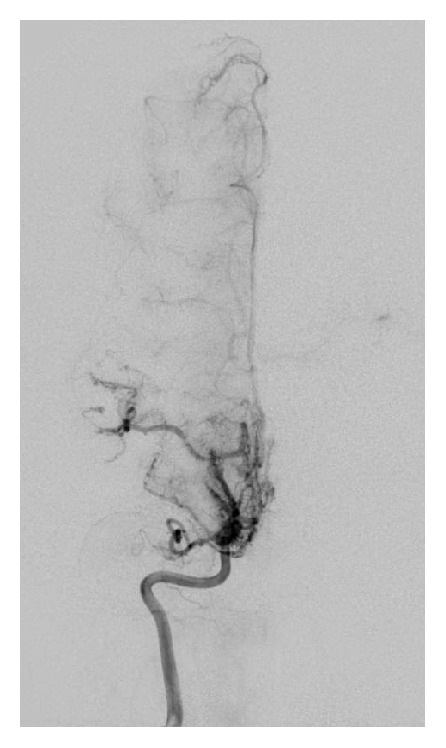
Right internal cerebral artery in cerebral angiogram.

**Figure 2:**
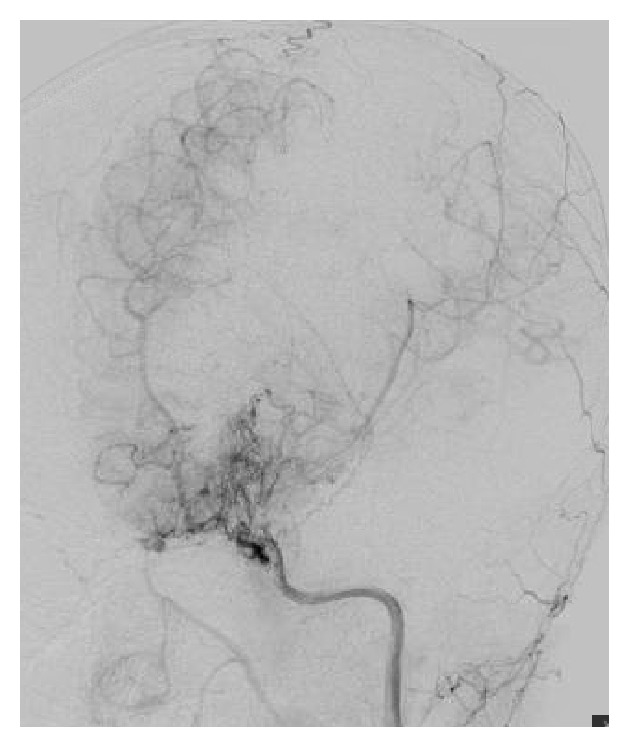
Left internal cerebral artery in cerebral angiogram.

**Figure 3:**
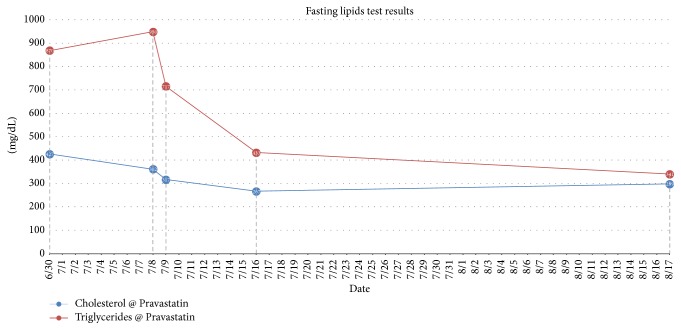
Graph showing decrease in cholesterol and triglycerides with Pravastatin treatment.

Patient is a 9-year-old previously healthy Caucasian female. She was adopted at 3 weeks of age and the family history is unknown. She has a past medical history of gross motor delay, followed closely by Neurology and Physical Therapy and was well by 3 years of age. The patient presented to the Emergency Department with an acute onset of right arm weakness, described as inability to move her arm and write. No history of recent trauma or illness. On arrival to the Emergency Department, vital signs obtained were BP 113/67, pulse 106, and temp. 36.7. Her weight was 26 kg (20% ile), height was 122 cm (3% cile), and BMI was 17 (70% ile). Physical exam is only significant for right arm motor weakness 3/5. No sensory deficit was noted. MRI done for stroke protocol revealed small occlusion of the distal internal carotid arteries and numerous small collateral vessels projecting from the circle of Willis region, and MRA was consistent with Moyamoya disease. Six-vessel angiogram confirmed the diagnosis with bilateral internal carotid arteries occlusion as shown in Figures 1 and 2. Baseline fasting lipid profile was abnormal: elevated fasting triglyceride (870 mg/dL, *n* < 150 mg/dL), elevated total cholesterol (427 mg/dL, *n* < 200 mg/dL), normal HDL 48 mg/dL, and LDL not measurable due to elevated triglycerides. Apolipoprotein B was elevated at 219 mg/dL (55 mg/dL–125 mg/dL). Thyroid function test was done and revealed slightly elevated TSH of 5.7 mcIU/mL (*n* 0.35 mcIU/mL–4.0 mcIU/mL) which was not considered clinically significant. She was placed on a pediatric low fat/low cholesterol diet based on nutrition recommendation. She was started on Pravastatin 20 mg daily. Seven days after initial presentation, she developed slurring of speech. MRI demonstrated new acute ischemic infarct involving the left frontal lobe, in the vascular territory of the anterior left middle cerebral artery. Prompt evaluation and revascularization were scheduled. Total cholesterol and triglyceride level decreased to 267 mg/dL and 433 mg/dL, respectively, after fourteen days of Pravastatin therapy, as demonstrated in Figure 3. Patient underwent bilateral superficial temporal artery-middle cerebral artery bypass (STA-MCA) with no complications.

## 3. Discussion

Moyamoya disease is an uncommon cerebrovascular disease characterized by progressive steno-occlusive changes in the terminal internal carotid arteries (ICA) and their main branches. A bimodal age distribution has been reported for Moyamoya, with a high peak at 5 years and at 40 years [3, 10, 11]. The clinical features of Moyamoya disease differ between children and adults. Moyamoya disease often presents in children initially as a stroke often accompanied by muscular weakness. This is in contrast to adults who typically present with subarachnoid or intraparenchymal hemorrhage [11, 12]. The predominance of ischemic events in childhood, but hemorrhagic strokes in adulthood, has been noted in Taiwanese with MMD [13] Diagnosis is usually possible with a magnetic resonance imaging (MRI) scan to look at the brain and a magnetic resonance angiogram (MRA) to look at the blood vessels of the brain. Although a genetic role has been postulated particularly due to higher incidence of Moyamoya and familial Moyamoya among Asians and Asian-Americans, the pathogenesis underlying this condition among other ethnicities and within North American populations is still unclear. Immunologic basis has been a study of interest due to association of Moyamoya with autoimmune diseases such as Graves' disease and Systemic Lupus Erythematous (SLE). Atherosclerosis and thyroid disease are the most frequent concurrent diseases with MMD especially on the adult population [1, 10].

Long-term prognosis for patients with nonsurgically treated MMD is not fully understood. However, some reports have described its natural clinical course and the results of conservative treatment. Kuroda et al. [14] reported a disease progression rate of approximately 20% over 6 years. Being female was identified as an independent risk factor for disease progression by multivariate analysis. Other investigations on the progression rate of the unaffected side of surgically treated unilateral MMD reported that six of the 41 cases (14.6%) exhibited contralateral progression during the mean follow-up of 34 months [15]. Considering these reports, MMD seems to have a progressive nature. Among many studies examining risk factors for MMD progression, the presence of thyroid disease such as Graves' disease has been a well-known medical condition linked to rapid progression of MMD [16, 17]. Recently, the RNF213 variant was suggested as a possible causative genetic alteration leading to the development as well as progression of MMD [18]. Because surgical revascularization has been recommended for symptomatic patients with impaired hemodynamics, some studies have described the outcomes of conservative treatment among asymptomatic or hemodynamically stable patients with MMD. A multicenter, nationwide survey for conservative treatment results was conducted in 2007 in Japan. The authors reported the annual stroke rate as 3.2% from the observation of 34 asymptomatic patients conservatively followed over 44 months. Hemodynamic disturbance was revealed to be a risk factor for newly developed stroke [19]. In a North American series, the rates of annual ischemic and hemorrhagic stroke rate were reported as 13.3% and 1.7%, respectively. Being female and smoking were risk factors for stroke development [20]. Cho et al. reported an annual stroke rate of 4.5% among 241 hemodynamically stable patients with MMD over 83 months. The annual stroke rate was higher in the hemorrhagic presentation group (5.7%) than the ischemic presentation group (4.2%) or the asymptomatic group (3.4%). They found familial disease and thyroid disease to be risk factors affecting stroke occurrence [21]. As for ischemic presenting MMD, 5.6% of the annual ischemic stroke rate also reported that posterior circulation involvement was a strong risk factor for ischemic stroke [22]. Antiplatelet treatment for preventing stroke in patients with MMD had been utilized by many physicians, especially in non-Asian areas. According to the reports of a worldwide survey, 31% of responders agreed to use long-term acetylsalicylic acid [23]. However, the evidence for antiplatelet treatment is lacking. Recently, the efficacy of antiplatelet therapy for preventing stroke was investigated in a cohort study with a large sample size. According to the authors, antiplatelet therapy could not prevent recurrent cerebral infarction for ischemic presenting patients with MMD. The nature of the ischemic insult in patients with MMD is not an embolic infarction, but instead it is mainly a hemodynamic infarction. The pathologic changes of the MMD vessels near the ICA bifurcation are not a type of endothelial damage, which is prone to platelet adhesion. Therefore, theoretically, antiplatelet drugs will not be effective for preventing ischemic stroke in patients with MMD. Although antiplatelet users are subject to hemorrhagic complications, the therapy was not associated with an increase in cerebral hemorrhage among patients with MMD [24]. Thus, prescribing antiplatelet agents for symptomatic patients with MMD should not yet be considered as an alternative treatment.

Pathologic analysis has demonstrated that affected vessels generally do not exhibit arteriosclerotic or inflammatory changes, even though there may be minimal lipid deposition seen in the intima with fibrous thickening [25, 26]. Rather, vessel occlusion results from a combination of both hyperplasia of smooth muscle cells and luminal thrombosis [10]. The Moyamoya collaterals are dilated perforating arteries believed to be a combination of preexisting and newly developed vessels [26, 27]. A number of growth factors, enzymes, and other peptides have been reported in association with Moyamoya, including basic fibroblast growth factor, transforming growth factor-*β*1, hepatocyte growth factor, vascular endothelial growth factor, matrix metalloproteinases, intracellular adhesion molecules, and hypoxia-inducing factor-1*α*, among others [27–34].

In children with primary hyperlipidemia, familial combined hyperlipidemia (FCHL) is expressed three times more commonly than familial hypercholesterolemia and half of the siblings are affected. In our patient, family history is not available. However, FCHL can be defined as elevated fasting cholesterol and triglycerides, with Apo B level of >120 mg/dL, which is consistent with the level seen in our patient. Prognosis includes development of cerebrovascular disease and insulin resistance leading to type 2 diabetes, which itself is a risk factor for atherosclerosis. Long-term prognosis of FCHL in a pediatric patient is not yet well understood as limited pediatric patients with FCHL have been followed through adulthood. Mainstay of treatment of any form of hypertriglyceridemia includes risk-factor control and diet. Children older than 2 years are usually managed with weight control and step-one diet, that is, less than or equal to 30% of total calories being fat [35]. One of the leading comorbidities documented in patients with Moyamoya is hyperlipidemia. However, no study has been reported regarding incidence of Moyamoya coexistent with hypertriglyceridemia on a pediatric patient. Recent case reports and meta-analysis involving patients with Moyamoya and concurrent hyperlipidemia all included patients over 30 years of age. These patients were not diagnosed to have familial hypercholesterolemia (FHC) nor familial combined hyperlipidemia (FCHL). Reasons for elevated cholesterol levels were not clear and were sometimes attributed to other occlusive vascular diseases, as well as untreated hypothyroidism, which is uncommon in the pediatric age group. Our patient by far is the first pediatric patient to have Moyamoya disease and concurrent FCHL, being well managed with Pravastatin.
